# Critical care services and the H1N1 (2009) influenza epidemic in Australia and New Zealand in 2010: the impact of the second winter epidemic

**DOI:** 10.1186/cc10266

**Published:** 2011-06-09

**Authors:** The ANZIC Influenza Investigators

## Abstract

**Introduction:**

During the first winter of exposure, the H1N1 2009 influenza virus placed considerable strain on intensive care unit (ICU) services in Australia and New Zealand (ANZ). We assessed the impact of the H1N1 2009 influenza virus on ICU services during the second (2010) winter, following the implementation of vaccination.

**Methods:**

A prospective, cohort study was conducted in all ANZ ICUs during the southern hemisphere winter of 2010. Data on demographic and clinical characteristics, including vaccination status and outcomes, were collected. The characteristics of patients admitted during the 2010 and 2009 seasons were compared.

**Results:**

From 1 June to 15 October 2010, there were 315 patients with confirmed influenza A, of whom 283 patients (90%) had H1N1 2009 (10.6 cases per million inhabitants; 95% confidence interval (CI), 9.4 to 11.9) which was an observed incidence of 33% of that in 2009 (*P *< 0.001). The maximum daily ICU occupancy was 2.4 beds (95% CI, 1.8 to 3) per million inhabitants in 2010 compared with 7.5 (95% CI, 6.5 to 8.6) in 2009, (*P *< 0.001). The onset of the epidemic in 2010 was delayed by five weeks compared with 2009. The clinical characteristics were similar in 2010 and 2009 with no difference in the age distribution, proportion of patients treated with mechanical ventilation, duration of ICU admission, or hospital mortality. Unlike 2009 the incidence of critical illness was significantly greater in New Zealand (18.8 cases per million inhabitants compared with 9 in Australia, *P *< 0.001). Of 170 patients with known vaccination status, 26 (15.3%) had been vaccinated against H1N1 2009.

**Conclusions:**

During the 2010 ANZ winter, the impact of H1N1 2009 on ICU services was still appreciable in Australia and substantial in New Zealand. Vaccination failure occurred.

## Introduction

Influenza A H1N1 2009 emerged in Mexico in early 2009 and spread rapidly causing a pandemic. The World Health Organization (WHO) declared a phase 6 influenza pandemic on 11 June 2009 and declared it to be over on 10 August 2010 [1]. The first wave of the H1N1 2009 outbreak was notable for the number of fatal cases among young people and atypical risk factors for developing severe diseases. Since 19 April 2009, WHO had reported over 491,766 laboratory-confirmed cases of H1N1 2009 and 18,449 related deaths [1].

People in Australia and New Zealand (ANZ) were significantly affected by the virus, with a total of 43,700 confirmed cases in Australia as of October 2010, with 6,064 cases occurring between 1 January and 15 October 2010 [2]. We have reported previously the serious impact of the virus on the provision of critical care services in 2009 [3]. In order to rapidly inform health professionals in the Northern Hemisphere this study censored new incident cases before the end of the influenza season. In 2010, the deployment of vaccination and the acquisition of natural immunity against H1N1 2009 were expected to decrease the burden of disease due to influenza [4]. Australia and New Zealand were among the first countries to experience a second influenza season with H1N1 2009 following widespread deployment of vaccination.

In this report, we describe the incidence of intensive care unit (ICU) admissions, demographic and clinical characteristics (including vaccination status) and outcome of all patients with laboratory confirmed H1N1 2009 admitted to ICUs in ANZ during the second winter (2010) of this influenza epidemic. We compare these characteristics with those of patients admitted to ICU during the corresponding period of 2009.

## Material and methods

We performed a multicentre study in 187 ICUs in ANZ comprising all adult, paediatric and combined adult and paediatric ICUs [5]. These ICUs had a total of 1,821 beds, of which 1,487 were equipped for mechanical ventilation. Each centre or region obtained Ethics Committee approval and the requirement for individual subject informed consent was waived at all sites. We report our findings according to STROBE guidelines for observational studies [6].

Between 1 June and 15 October, in both 2009 and 2010, we screened for patients admitted to ICU with confirmed influenza A. Influenza A was confirmed by reverse transcriptase polymerase chain reaction (RT-PCR), antigen detection, or serology. H1N1 2009 and seasonal subtypes, H1N1 and H3N2, were determined by RT-PCR or specific serology. The laboratories were accredited by the National Association of Testing Authorities in Australia or by International Accreditation New Zealand. Population data for Australia and New Zealand were obtained from Australian Bureau of Statistics [7] and Statistics New Zealand [8] for 2009 and 2010.

We collected patient-specific data as described previously [3], although vaccination status against H1N1 2009 virus was collected only during 2010. We divided patients into the age groups used in a previous report [9]. We calculated the duration of ICU and hospital stay and ICU occupancy rates for Australia and New Zealand. We recorded patient outcomes at ICU and hospital discharge status or as still in hospital or in ICU as of 15 October 2010 for patients admitted in 2010 and as of 23 November 2009 for patients admitted in 2009. Finally, we obtained data on vaccination in both countries.

### Data management and statistical methods

We collected data using electronic case report forms. The study-coordinating centre was the Australian and New Zealand Intensive Care-Research Centre, Monash University, Melbourne, Australia [10]. H1N1 2009 infection is subject to mandatory reporting in both Australia and New Zealand and wherever possible diagnoses were confirmed with the relevant public health authorities. In addition, to confirm the completeness of case ascertainment, we contacted all ICUs that had no reported cases at the end of each study period. Cases transferred between ICUs were counted as a single ICU case. We made no assumptions for missing data and all proportions were calculated as percentages of available data.

We performed statistical analysis using SAS version 9.1 (SAS Institute Inc., Cary, NC, USA). We calculated descriptive statistics for all study variables. We report continuous variables as medians with interquartile range (IQR) and categorical variables as percentages with 95% confidence interval (95% CI) where appropriate. We estimated age-based population admission rates [7,11]. We compared binomial variables of the second winter (2010) with those of the first winter (2009) using Chi-square tests for equal proportion or Fisher's Exact test where numbers were small. Comparisons between continuous variables were made using Wilcoxon rank sum test. A two-sided *P*-value of < 0.05 was considered to be statistically significant.

## Results

During the study period, from 1 June until 15 October 2010, 315 patients with confirmed influenza A were admitted to an ICU, compared with 1,113 patients during the same corresponding period in 2009. The predominant sub-type was H1N1 2009 in both years (90% in 2010 versus 83% in 2009, *P *= 0.002). The distribution of sub-types of influenza A is reported in Table 1. In 2010 there were 283 patients with confirmed H1N1 2009 admitted to ICU, corresponding to a population incidence of admission to ICU of 10.6 (95% CI, 9.4 to 11.9) per million inhabitants [7,8]. By comparison, during 2009 there were almost three times as many patients with confirmed H1N1 2009 (n = 921), corresponding to a population incidence of admission to ICU of 35.2 (95% CI, 32.9 to 37.5) per million inhabitants [7,8] (*P *< 0.001). The geographical distribution of admissions was also different between 2010 and 2009 (Table 1). In 2010 the population incidence in Australia was 2-fold lower than in New Zealand (*P *< 0.001), whereas in 2009 this relationship was reversed with a 1.3-fold higher incidence in Australia (*P *= 0.009). The overall incidence, combining 2009 and 2010, was 45.3 (95% CI, 42.4 to 48.2) admission to ICU per million inhabitants in Australia and 47.1 (95% CI, 40.5 to 53.7) in New Zealand (*P *= 0.65).

**Table 1:** Epidemiological characteristics of ICU admissions due to influenza A in 2010 and 2009 in Australia and New Zealand

	2010	2009	*P-*value
**Number of Influenza A cases**	315	1113	
H1N1 2009	283 (90%)	921 (83%)	0.002
Seasonal H1N1	0 (0%)	36 (3%)	< 0.001
Seasonal H3N2	3 (1%)	24 (2%)	0.17
Not sub-typed or other	29 (9%)	132 (12%)	0.22
**Incidence of admission to ICU per million inhabitants**, (95% CI)			
ANZ	10.6 (9.4 to 11.9)	35.2 (32.9 to 37.5)	< 0.001
Australia	9 (7.8 to 10.3)	36.5 (34 to 39.1)	< 0.001
New Zealand	18.8 (14.6 to 23)	28.3 (23.2 to 33.5)	0.005
**Maximum daily ICU occupancy per million inhabitants**, (95% CI)	2.4 (1.8 to 3.0)	7.5 (6.5 to 8.6)	< 0.001

ANZ, Australia and New Zealand; ICU, intensive care unit

**Figure 1:**
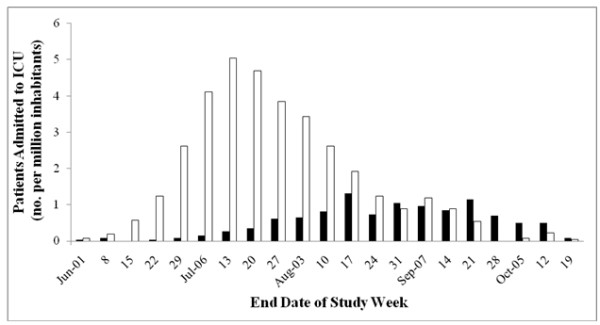
**Number ICU admission with H1N1 2009 according to study week during 2009 (white bars) and 2010 (dark bars)**.

The number of patients admitted to an ICU with H1N1 2009 according to study week is displayed in Figure 1. In 2010 the onset of the epidemic was delayed (by five weeks) and the peak incidence was lower. The impact on ICU services was significantly lower in 2010 with peak daily ICU bed occupancy being 7.5 (95% CI, 6.5 to 8.6) per million inhabitants in 2009 compared with 2.4 (95% CI, 1.8 to 3.0) per million inhabitants in 2010 (*P *< 0.001) (Table 1).

The clinical characteristics, risk factors, and outcomes of patients admitted to ICUs with H1N1 2009 were broadly similar in 2010 to those admitted in 2009 (Table 2). Comparing 2010 with 2009 there were no significant differences in the distribution of cases among different age groups. In both study periods the highest number of ICU admissions occurred among patients aged between 25 and 49. There was no difference in the proportion of patients with a body mass index greater than 35 kg/m^2^, who were pregnant or post-partum or who had no known predisposing factor between that observed in 2009 and 2010 (Table 2). The proportion of patients with asthma or chronic obstructive pulmonary disease, chronic heart failure, or an Acute Physiology Age Chronic Health Evaluation (APACHE) III or paediatric co-existing illness was lower in 2010, but these factors were still over-represented in comparison to the general population, as they were in 2009 [3] (Table 2). Furthermore, a similar spectrum of clinical illness was observed in 2010 as in 2009, with viral pneumonitis or the acute respiratory distress syndrome (ARDS) being the most common presentation. The severity of illness, as inferred from the proportion of patients treated with mechanical ventilation, renal replacement therapy and vasopressor medications, was not different between the two study periods. The durations of admission to ICU and to the hospital were 8.5 (IQR = 3.2 to 16.1) days in 2010 compared with 7.0 (IQR = 3.0 to 16.0) days in 2009 (*P *= 0.32), and 15.4 (IQR = 7.5 to 25.6) in 2010 compared with 16.6 (IQR = 7.0 to 31.0) in 2009 (*P *= 0.38), respectively. In addition, in 2010, 13% of patients died before hospital discharge. This proportion was not different to that observed in 2009, although follow up was more complete for patients in 2009, with 45 patients (15.9%) from 2010 still being in hospital at time of censoring compared with 63 patients (6.8%) in 2009.

**Table 2:** Characteristics of patients, clinical presentation and outcome of confirmed critical illness related to H1N1 2009 influenza in 2010 and 2009

	2010	2009	*P*-value
**Age, years**			
Median (IQR)	42 (28 to 53)	39.8 (27 to 53)	0.86
**Age distribution, years **no./total no. (%)			0.13
0 to < 1	8/271 (3%)	34/921 (4%)	0.45
1 to 4	12/271 (4%)	21/921 (2%)	0.1
5 to 24	31/271 (11%)	128/921 (14%)	0.21
25 to 49	128/271 (47%)	415/921 (45%)	0.92
50 to 64	87/271 (32%)	242/921 (26%)	0.13
> 65	16/271 (6%)	79/921 (9%)	0.11
**Female sex**, no./total no. (%)	134/283 (47%)	498/921 (54%)	0.048
**Pregnant or post-partum women**,	29/283(10.2%)	81/921 (8.8%)	0.54
**Ethnicity**, no./total no. (%)			
Caucasian	212/280 (75.7%)	633/887 (71.4%)	0.16
Aboriginal or Torres Strait Islander admitted to ICU in Australia	9/199 (4.5%)	82/765 (10.7%)	0.01
Maori admitted to ICU in New Zealand	16/81 (19.8%)	34/122 (27.9%)	0.25
Pacific Islander	13/280 (4.6%)	43/887 (4.8%)	0.89
Asian	13/280 (4.6%)	39/887 (4.4%)	0.86
Other	17/280 (6%)	56/887 (6.3%)	0.88
**Adults with BMI > 35**, no./total no. (%)	60/215 (27.9%)	177/642 (27.6%)	0.96
**Diabetes**, no./total no. (%)	42/277 (15%)	148/900 (16%)	0.61
**Asthma or chronic obstructive pulmonary disease**, no./total no. (%)	73/278(26%)	297/915 (32.5%)	0.05
**Chronic heart failure**, no./total no. (%)	14/277 (5%)	99/910 (10.9%)	0.004
**APACHE III or pediatric co-morbidity**, no./total no. (%)	52/279 (19%)	251/891 (28.2%)	0.002
**No known predisposing factors**, no./total no. (%)	107/283 (38%)	300/921 (33%)	0.1
**Influenza syndrome**			< 0.001
Viral pneumonitis or ARDS, no./total no. (%)	147/255 (58%)	438/896 (48.9%)	0.014
Secondary bacterial pneumonia, no./total no. (%)	71/255 (28%)	177/896 (19.6%)	0.006
Exacerbation of airflow limitation, no./total no. (%)	20/255 (8%)	120/896 (13.4%)	0.017
Inter-current illness or other, no./total no. (%)	17/255 (6.7%)	161/896 (18%)	0.001
**Duration of treatment in ICU, days** Median (IQR)	8.5 (3.2 to 16.1)	7 (3.0 to 16)	0.32
**Duration of treatment in hospital, days** Median (IQR)	15.4 (7.5 to 25.6)	16.6 (7.0 to 31.0)	0.38
**Patients treated with MV**, no./total no. (%)	186/282 (66%)	579/909 (63.7%)	0.53
**Patients treated with vasopressor drugs**, no./total no. (%)	104/269 (39%)	276/787 (35%)	0.29
**Patients treated with renal replacement therapy**, no./total no. (%)	20/275 (7.3%)	50/800 (6.3%)	0.65
**Patients treated with ECMO**, no./total no. (%)	12/278 (4.3%)	57/579 (9.8%)	0.008
**Mortality**, no./total no. (%)	32/238 (13%)	136/858 (15.9%)	0.42

ARDS, acute respiratory distress syndrome; APACHE III, Acute Physiology Age Chronic Health Evaluation III; BMI, body mass index; ECMO, extra corporeal membrane oxygenation; ICU, intensive care unit; MV, mechanical ventilation

A vaccine for H1N1 2009 was not available at any time during the 2009 study period but became available in Australia and New Zealand between the two study periods. Publically available data suggest that at least 4.86 million (21.8%) Australians [12] and 1.05 million (24.1%) of New Zealanders (personal communication, Jane Chambers, Ministry of Health) were vaccinated against H1N1 2009 up to the end of June 2010. Of 170 patients admitted in 2010 for whom vaccination status was known, 26 (15.3%) had been vaccinated against H1N12009, of whom 16 were from Australia and 10 from New Zealand.

## Discussion

We studied all patients with confirmed H1N1 2009 infection admitted to Australian and New Zealand ICUs during the second winter of the epidemic in 2010 and compared this experience with that which occurred during the winter of 2009. We identified 283 patients with confirmed H1N1 2009 infection, giving a population incidence of 10.6 ICU admissions per million inhabitants, a quarter of the incidence observed in 2009. H1N1 2009 remained the dominant cause of ICU admissions due to confirmed influenza in both countries [3]. The second wave occurred later in the year but resulted in similar illness severity, affected similar groups at risk and caused similar in-hospital mortality. The incidence of H1N1 2009 influenza critical illness during 2010 was higher in NZ than in Australia, which was a reversal of the pattern in 2009. However, the combined incidence over both winters was similar in both countries. We observed vaccination failure in a substantial proportion of patients for whom vaccination status was known. Our observations do not support the view that the H1N1 2009 pandemic has come to an end [13].

Although the incidence of critical illness was significantly lower in 2010 the pattern of illness among patients who were admitted to an ICU was similar to that observed in 2009 and in other reports of patients admitted to ICUs [14-16]. ARDS was present in more than 50% of patients, as previously described in ANZ [3] and worldwide [14,15,17-21]. The length of stay in ICU was unchanged [3] and similar to that reported elsewhere [14,20]. The risk factors for admission to ICU were similar to those reported during the first wave [3,22,23]. In addition, the treatments administered and the mortality that occurred were similar to that observed in 2009 in ANZ as well as elsewhere [3,13,14,18].

The incidence of ICU admission per million inhabitants was higher in Australia than in New Zealand in 2009 (36.5 versus 28.3 admissions to ICU per million inhabitants) [3]. Conversely, in 2010 this incidence was higher in New Zealand. This difference is concordant with national data reporting 6,064 confirmed H1N1 cases in Australia (272 cases per million inhabitants) versus 1,810 in New Zealand (415 cases per million inhabitants in 2010) during the same period [2,24]. The proportion of confirmed cases hospitalised (727 of 1,810 = 40.1%) [25], and of hospitalised cases admitted to ICU (82 of 732 = 11.2%) in New Zealand was similar to 2009 [26]. Accordingly, the higher ICU admission rate per population observed in 2010 was due to a higher incidence of community infection, rather than a difference in severity of disease. The reason for the difference in infection rate between Australia and New Zealand in 2010 is unclear, as a similar proportion of the population was seropositive after the 2009 pandemic wave in Australia (22% (95% CI 19.1 to 24.9)) compared to New Zealand (26.7% (95% CI 22.6 to 29.4)) [27,28] and a similar proportion were vaccinated (21.8% versus 24.1%, respectively) during the inter-wave period. Possible explanations include natural variations in community spread of influenza or an effect of the difference in vaccination deployment where Australia used both early monovalent and later polyvalent vaccines while New Zealand relied predominantly on delivery of the polyvalent vaccine alone, although overall coverage was similar.

We found that 15.3% of those patients for whom data were available had been previously vaccinated, which is consistent with another report of H1N1 2009 vaccination effectiveness [29]. In a critically ill patient with a severe pneumonia and a history of H1N1 vaccination the possibility of vaccination failure and H1N1 2009 infection should be considered.

Our data are subject to some limitations. To make this report available in a timely manner, we censored hospital outcome data. Ascertainment of cases of H1N1 2009 admitted to ICUs in 2010 as well as in 2009 may not have been complete, and we cannot exclude the possibility that a small number of cases were not reported to the registry, and false negative diagnostic tests may well have underestimated the true burden of H1N1 2009 in our patients. Among the patients with confirmed influenza A, there were 29 in whom the influenza was not sub-typed in 2010 and 132 in 2009. Thresholds for undertaking testing both in hospital and in the community were not standardised. It is not possible to reach a conclusion regarding the efficacy of vaccination as the vaccination status of many patients was unknown. Finally, while we report a similar severity of the illness to 2009, we do not have information about anti-viral treatment, potential viral mutation and resistance to anti-viral drugs. Finally, we did not evaluate the role played by a corticosteroid therapy on the outcome of patients with ARDS, and we were not able to cope with the controversy about the effect of this treatment [30].

## Conclusions

In conclusion, the impact of the second H1N1 2009 winter epidemic was still substantial although significantly less than in 2009. It had a lower peak occurring approximately five weeks later than in 2009, affected similar individuals, was similar in clinical severity, carried a similar mortality rate. In patients with H1N1 2009 infection requiring ICU admission a number of apparent vaccination failures were observed. Based on these data and despite the deployment of the vaccination, a second season of H1N1 2009 influenza may still have a substantial intensive care impact.

## Key messages

• H1N1 (2009) had a substantial impact on ICU resources during the winter of 2010 in Australia and New Zealand.

• Risk factors remain similar to those reported in 2009 and include obesity, pregnancy and presence of comorbidity.

• In 2010, the illness severity, reflected by treatment with mechanical ventilation, renal replacement therapy, vasopressor drug, and extracorporeal membrane oxygenation as well as by hospital mortality, was similar to that observed the previous year.

• Vaccination against H1N1 (2009) doesn't exclude the possibility of developing critical illness due to infection with H1N1 (2009) flu.

## Abbreviations

ANZ: Australia and New Zealand; ARDS: acute respiratory distress syndrome; ECMO: extracorporeal membrane oxygenation; ICU: intensive care unit; RT-PCR: reverse transcriptase polymerase chain reaction.

## Competing interests

This study was supported by the Department of Health and Ageing, Commonwealth Government of Australia; New South Wales Health, Government of New South Wales; Department of Health, Government of Victoria; the Australian and New Zealand Intensive Care Research Centre; the Australian and New Zealand Intensive Care Society; and an unrestricted grant from CSL Limited, Melbourne, Victoria.

## Authors' contributions

All authors have contributed to all components
